# Diabetic Coma Symptoms, Pathology and Treatment

**Published:** 1902-04

**Authors:** 


### Diabetic Coma Symptoms, Pathology and Treatment.

Abraham Mayer, *New York Medical Record,* lays stress upon the presence of Kussmaul's "air hunger" respiration, and of the hyaline or granular casts of Kültz prior to all attacks of diabetic coma, while large and increasing amounts of beta-oxybutyric and diacetic acids with acetone and ammonia are invariably present, often for some time before the attack.

According to the acid intoxication theory the various acids found combine with the alkalies of the body after proteid foods have ceased to furnish enough to maintain the balance, this condition being rapidly followed by a true acid intoxication and coma. The advocates of the toxic theory, however, claim that these acids destroy protoplasm directly generating toxins.

As facts have been found to increase the acetone and diacetic acids generated, besides radically disturbing the digestion, while proteids directly favor the production of toxins, it is well to allow carbohydrates in limited quantities, while alkalies should be freely administered.

The author cites a case in which two attacks of coma were successfully treated by 70 and 50 grams of glycuronic acid, respectively, the patient succumbing to a third attack after the supply of the acid was exhausted. As Uso tropin splits into ammonia and formaldehyde in the presence of an acid, it forms a rational treatment, and was used with much satisfaction in a second case.

